# Introduction to what we still need to learn about ARIA

**DOI:** 10.1002/alz.085571

**Published:** 2025-01-03

**Authors:** Reisa A Sperling

**Affiliations:** ^1^ Brigham and Women’s Hospital, Harvard Medical School, Boston, MA, USA; Center for Alzheimer’s Research and Treatment, Brigham and Women’s Hospital/Harvard Medical School, Boston, MA USA

## Abstract

The short introduction will highlight what we have learned about amyloid related imaging abnormalities (ARIA) since 2011 and the critical questions that remain to be elucidated. In particular, we need to better understand the risk factors and mechanisms that underlie the relatively rare symptomatic cases. We will highlight the importance of bi‐directional translational research to bring insights from the laboratory to the clinic and findings from clinical trials back to the bench.

